# Spatial Reasoning and Its Contribution to Mathematical Performance Across Different Content Domains: Evidence from Chinese Students

**DOI:** 10.3390/jintelligence13040041

**Published:** 2025-03-24

**Authors:** Tianshu Xu, Siyu Sun, Qiping Kong

**Affiliations:** 1 School of Educational Science, Nantong University, Nantong 226019, China; 2 College of Elementary Education, Capital Normal University, Beijing 100048, China; 3 Faculty of Education, East China Normal University, Shanghai 200062, China; qpkong@kcx.ecnu.edu.cn

**Keywords:** spatial reasoning, mathematical performance, Chinese students

## Abstract

Recent studies have provided convincing evidence highlighting the strong relationship between spatial reasoning and mathematical performance. However, there is a limited body of research exploring the contributions of different spatial reasoning constructs to mathematical performance across various content domains, particularly within non-Western contexts. This study investigates the relationship between spatial reasoning skills—including mental rotation, spatial visualization, and spatial orientation—and mathematical performance across various domains (number, geometric shapes and measures and data display) among Chinese elementary school students in grade four (ages 9–10). The results indicate that overall spatial reasoning significantly predicts mathematical performance across various domains. All three spatial reasoning constructs significantly contribute to performance in the number and geometric shapes and measures domains, with mental rotation and spatial orientation being the strongest predictors of performance in these respective content domains. For data display performance, spatial orientation and spatial visualization significantly contribute, with spatial visualization being the strongest predictor. Although no significant gender differences were found in the overall link between spatial reasoning and mathematical performance, subgroup regression analysis showed variations. For male students, spatial orientation was the main predictor across content areas. For female students, mental rotation was the key predictor for number and geometry, while spatial visualization was most significant for data display.

## 1. Introduction

Spatial reasoning has garnered increasing attention from researchers in education and psychology due to its critical role in students’ academic performance in STEM subjects and their future engagement in STEM careers (Lubinski 2010; Uttal et al. 2013; Wai et al. 2009). With increasing evidence highlighting the significant correlation between spatial reasoning and mathematical performance (Gunderson et al. 2012; Mix et al. 2016; Verdine et al. 2017; Frick 2019), considerable efforts have been directed toward enhancing students’ math skills through spatial training (Hawes et al. 2017; Lowrie et al. 2017, 2018, 2019, 2021). However, not all positive effects of spatial training have successfully transferred to mathematical performance (Hawes et al. 2015; Cornu et al. 2017). This may be due to the unclear relationship between different spatial reasoning constructs and their varying impacts on mathematical performance across different content domains, including both geometric and non-geometric areas. Cross-cultural comparative research shows that, compared to several Asian mathematics curricula which have put more emphasis on geometry, space appears to have received higher curricular esteem in Western contexts, such as in Australia (Lowrie et al. 2016). The conception and practice of spatial reasoning differ across cultural contexts. Considering that students from East Asian countries typically perform above the international average in mathematics and science assessments (OECD 2014, 2016, 2019), there is also a need for research in non-Western contexts, such as China, to provide a more comprehensive understanding of this relationship. Moreover, despite the growing recognition of spatial reasoning’s role in mathematical learning, there remains a clear need for further psychometric analyses of spatial reasoning measures (Uttal et al. 2024). Robust psychometric evaluation is crucial for accurately capturing students’ spatial abilities and their relationship with mathematical performance. To address these gaps, this study aimed to validate the modified spatial reasoning test for quality and applicability among Chinese students in grades 4 to 6 (ages 9–12), and to further investigate the nuanced relationships between spatial reasoning constructs and mathematical performance across different content domains among 4th grade students (ages 9–10).

### 1.1. Spatial Reasoning Constructs

Spatial reasoning has been a topic of significant research interest over a long period, yet its complexity has led to a lack of consensus on its precise definition and components. Nevertheless, it is widely recognized that spatial reasoning encompasses multiple sub-skills rather than being a singular capability. McGee (1979) was one of the earliest researchers to identify two primary components of spatial reasoning: spatial visualization and spatial orientation. Spatial visualization involves the mental manipulation of objects, including imagining rotations, twists, and transformations. Spatial orientation involves understanding the arrangement of elements within visual stimuli, not being confused by changes in direction, and recognizing spatial positions relative to one’s body. Linn and Petersen (1985) later expanded this understanding, categorizing spatial ability into three factors: spatial perception, mental rotation, and spatial visualization. They distinguished mental rotation from spatial visualization, considering them separate constructs. Spatial visualization may involve processes from spatial perception and mental rotation but requires multi-step analytical strategies, making it distinct from the other two. Tartre (1990) expanded on McGee’s work and further subdivided spatial visualization into two distinct factors: mental rotation (manipulating entire objects) and mental transformation (manipulating parts of objects).

Over the past decade, spatial orientation and spatial visualization have consistently been recognized as two primary spatial constructs (Clements and Sarama [2009] 2014). Additionally, some studies identify mental rotation as an independent spatial construct, distinct from spatial visualization. In this context, Ramful et al. (2017) advanced the field by proposing a three-tier framework of spatial reasoning that encompasses mental rotation, spatial orientation, and spatial visualization. This framework was developed through an analysis of tasks from the primary and lower secondary mathematics curricula. Building on this framework, the Spatial Reasoning Instrument, consisting of multiple tasks, was developed specifically designed to aid teachers and researchers in educational settings. Their work is notable for its emphasis on the practical application of spatial reasoning within schools, distinguishing it from other studies that may be more theoretical or conducted in controlled environments. This focus on the educational context provides a unique perspective on spatial reasoning, bridging the gap between theoretical constructs and their application in real-world educational settings.

Definitions and components of spatial reasoning differ depending on the discipline and focus of the study. Despite these variations, the present study is specifically concerned with spatial reasoning as it manifests in educational contexts, particularly within the framework of school-based learning. Accordingly, this research adopts the spatial reasoning framework proposed by Ramful et al. (2017), which offers a robust and education-oriented approach. Their delineation of spatial reasoning into three distinct constructs—mental rotation, spatial orientation, and spatial visualization—provides a comprehensive structure that is particularly relevant for evaluating students’ spatial reasoning skills in the context of primary and secondary education. The subsequent sections will offer an in-depth examination of each of these three spatial constructs.

#### 1.1.1. Mental Rotation

Mental rotation is the ability to accurately rotate a two-dimensional (2D) shape or a three-dimensional (3D) object in the mind (Lowrie et al. 2018). During this process, the object’s internal structure remains unchanged, and the observer’s perspective stays constant. This ability is typically assessed through tasks that require individuals to identify or match identical figures presented at different orientations, such as the comparison tasks devised by Shepard and Metzler (1971).

#### 1.1.2. Spatial Orientation

Spatial orientation refers to the ability to reconfigure one’s position within space, involving the process of understanding spatial relationships from various scales, perspectives, and contexts. Unlike mental rotation, which involves object-centered transformations, spatial orientation is characterized by egocentric transformations (Hegarty and Waller 2005; Kozhevnikov and Hegarty 2001). This means it is centered on the observer’s perspective, requiring them to imagine spatial changes from their own viewpoint and incorporate perspective shifts, where the observer visualizes an object or scene from different orientations. In spatial orientation tasks, individuals must position themselves at a specified location, determine spatial relationships with other objects from that position, assess the object’s state or movement, and identify its final position after movement.

#### 1.1.3. Spatial Visualization

Spatial visualization is the ability to mentally manipulate or transform the visual and spatial properties of objects (Lowrie et al. 2019). It is the most complex of the three spatial reasoning constructs. Unlike mental rotation and spatial orientation, which involve treating the object as a whole during transformations, spatial visualization encompasses intricate internal changes within the object (Sorby 1999). These transformations can alter the object’s state or structure, such as unfolding a three-dimensional shape into a two-dimensional figure, folding a two-dimensional net into a three-dimensional shape, performing reflections, decomposing a composite figure into parts, or engaging in activities like origami and paper cutting.

### 1.2. Links Between Spatial Reasoning and Mathematical Performance

Spatial reasoning has long been recognized as a critical component in the development of mathematical understanding. However, the relationship between spatial thinking and mathematics is not straightforward (Clements and Sarama [2009] 2014). One common theoretical perspective is that mathematical thinking is supported by spatial representations (Hegarty and Kozhevnikov 1999; Mix and Cheng 2012). In this context, spatial representations function as cognitive tools enhancing the understanding of relative magnitude, measurement units, and arithmetic operations (Cipora et al. 2015; Congdon et al. 2018a; Newcombe et al. 2018). On the “mental blackboard” of spatial representation, various mathematical concepts, relationships, and operations are allowed for modeling and visualization (Lourenco et al. 2018; Mix 2019). Alternatively, it is argued that mathematics is inherently spatial. For instance, symmetry is not only a fundamental concept in geometry but also a core attribute of mathematical thinking (Sinclair 2004). In learning about equations, symmetry provides a perspective for interpreting the equal sign beyond its role as a procedural step in calculations (Verdine et al. 2017; Patahuddin et al. 2018). Furthermore, the number line is a clear spatial representation of how children conceptualize integers (Gunderson et al. 2012). Additionally, neuroscientific research indicates that similar neural pathways are engaged when processing both spatial and numerical information (Walsh 2003; Hubbard et al. 2005), suggesting a shared cognitive process between these domains.

There is substantial empirical evidence demonstrating that spatial skills are linked to individual differences in math knowledge. Cheng and Mix (2014) found that training in mental rotation improved students’ performance on missing-term problems (e.g., 4 + __ = 12). An analysis of data from 804 sixth grade students in Singapore revealed significant statistical differences in six mathematical domains, including whole numbers, algebraic patterns, data and probability, and geometry and measurement, between students with high and low visuospatial levels (Logan 2015). Gilligan et al. (2019) explored the developmental relationship between spatial ability and mathematical achievement in children aged 6–10, demonstrating that spatial ability remained a significant predictor of math performance even after controlling for other known predictors. Moreover, several longitudinal studies have shown that spatial ability at one age predicts mathematical performance at a later age. For example, Gunderson et al. (2012) demonstrated that 5-year-olds’ mental rotation skills predicted their approximate calculation abilities at age 8, suggesting that spatial ability helps children acquire linear spatial representations of numbers, which in turn facilitates the development of their numerical knowledge. This study was one of the earliest to investigate the potential developmental mechanisms underlying the relationship between spatial ability and mathematical achievement, and it has since been followed by numerous longitudinal studies exploring this relationship from various perspectives and across different age groups (Verdine et al. 2014; Casey et al. 2015; Lauer and Lourenco 2016; Gilligan et al. 2017; Frick 2019). Furthermore, factor analysis studies suggest that spatial and mathematical abilities are separate but highly correlated (Hawes et al. 2019), and children with strong spatial skills consistently perform better on math achievement tests from kindergarten through to the end of elementary school, even when controlling for the influence of language abilities (Mix et al. 2016, 2017).

Despite extensive empirical evidence supporting a strong correlation between spatial skills and mathematical performance, the transfer of benefits from spatial training to improved math outcomes has not consistently met expectations. While several long-term interventions focused on spatial reasoning have demonstrated significant improvements in math performance following the completion of the training programs (Hawes et al. 2017; Lowrie et al. 2017, 2018, 2019, 2021), other studies have observed that although students’ spatial abilities were enhanced through spatial training, these improvements did not consistently translate into better mathematical achievement (Hawes et al. 2015; Cornu et al. 2017). Among these intervention studies showing a successful transfer effect, the program designs predominantly centered on geometric content. However, research indicates that the importance of spatial reasoning extends beyond the realm of geometry and is crucial for various mathematical tasks (Mix 2019). Therefore, it is crucial to explore the relationship between spatial reasoning and mathematics more comprehensively, including various spatial structures and mathematical content domains.

### 1.3. Present Study

As described, the fine-grained relationship between different spatial reasoning constructs and their varying impacts on mathematical performance across different content domains remains unclear. This lack of clarity may contribute to the mixed evidence regarding the extent to which improvements in spatial skills translate into better mathematical performance. Rather than making assumptions about these relationships, our study seeks to explore and provide further insights into them. Given the exceptional performance of Chinese students in international mathematics assessments, exploring the spatial reasoning abilities of Chinese students is crucial for a more comprehensive understanding of the relationship between spatial reasoning and mathematical performance. The Chinese education system, deeply rooted in Confucian culture, places a high value on academic achievement and task persistence (Stankov 2010). However, as in other Asian mathematics curricula, space has received less emphasis in Chinese mathematics curricula compared to Western contexts. This cultural context provides a distinctive research perspective that could offer a more comprehensive understanding of how spatial reasoning contributes to mathematical performance. It is essential to assess whether, and to what extent, spatial reasoning remains a significant predictor of mathematical performance in this specific educational context, as it can provide a more comprehensive understanding of how spatial reasoning supports mathematical learning across diverse curricular frameworks.

The overarching goal of the study is to further understand the nuanced relationships between spatial reasoning and mathematical performance across different content domains. Based on the substantial body of research evidence previously reviewed on the links between spatial reasoning and mathematical performance, this study aims to further explore this relationship without presupposing specific outcomes, and additionally address the following research questions:

1. To what extent does spatial reasoning continue to predict mathematical performance among Chinese students who have had relatively limited exposure to space-related curriculum content?
2. How do different constructs of spatial reasoning (mental rotation, spatial visualization, and spatial orientation) specifically impact mathematical performance in various content domains (number, geometric shapes and measures, and data display) among Chinese elementary school students?

## 2. Materials and Methods

### 2.1. Design

This study is structured into two phases. The first phase, a preliminary analysis, aims to validate the modified spatial reasoning test for quality and applicability among Chinese students in grades 4 to 6 (ages 9–10). As emphasized in a recent review on how to best assess spatial skills, there is a clear need for more psychometric analyses of spatial thinking measures, and adapted tests require reporting the basic psychometric properties to ensure their reliability and validity (Uttal et al. 2024). This phase addresses these concerns by rigorously evaluating the psychometric properties of the spatial reasoning test, thereby laying a solid foundation for the study findings. The second phase, the main study, examines the predictive relationship between spatial reasoning and mathematical performance through a battery of tests administered among Chinese elementary students in grade 4 (ages 9–10), focusing on how spatial reasoning influences students’ outcomes across various mathematical content domains.

### 2.2. Procedure and Participants

Four elementary schools located in three cities of China, namely Suzhou, Hangzhou, and Shenzhen, were involved in this study. These schools varied in educational quality, which helped to ensure the diversity of our sample. Data collection was conducted following ethical approval. Participation was voluntary, with verbal informed consent obtained from all students. No incentives were provided for participation.

The participants in the first phase of the study were 477 students (256 boys and 221 girls) from grades 4, 5, and 6, aged between 9 and 12. We focused on this age range because the Spatial Reasoning Instrument (Ramful et al. 2017), which served as a reference for our modified spatial reasoning test, was developed specifically for elementary school students in this age group (Lowrie et al. 2019). One class per grade was selected from each school. In all, 12 intact classes of students completed the spatial reasoning test in their own classrooms to ensure a range of academic ability levels within the respective schools. The tests were administered by classroom teachers within a 40 min session. The final sample consisted of 477 valid responses from the 486 collected test papers, including 158 fourth graders, 158 fifth graders, and 161 sixth graders.

The participants for the second phase, the main study, involved 816 fourth grade students (432 boys and 384 girls), aged 9 to 10. This focus on fourth graders was due to availability, time constraints, and other limitations within the four schools, as well as the consideration that the mathematics test we adopted was typically aimed at fourth grade students. Data collection for the main study occurred one month after the first phase, with none of the classes or students overlapping between the two phases. One class per grade was selected from each school, ensuring a diverse range of academic abilities. A total of 21 intact classes completed both the spatial reasoning and mathematics tests in their own classrooms. From the collected 875 spatial reasoning tests and 872 mathematics tests, the final sample consisted of 816 students who provided valid responses for both tests.

### 2.3. Materials

#### 2.3.1. Spatial Reasoning Test

The spatial reasoning test was developed based on the three-tier framework proposed by Ramful et al. (2017), and adjustments were made to the specific subject and corresponding content of each spatial construct. To align with the modified theoretical framework, 28 items from the Spatial Reasoning Instrument (Ramful et al. 2017) were retained, and 4 additional items were adapted from the Thinking About 3D Shapes test (Owens 2001) to ensure comprehensive coverage of each construct subject. Specifically, the test comprises 9 mental rotation tasks, 10 spatial orientation tasks, and 13 spatial visualization tasks. To ensure the content validity of the spatial reasoning test, six experts in mathematics education evaluated the test content using a structured questionnaire. The questionnaire introduced the test framework and asked experts to assess whether the items accurately measured elementary students’ spatial reasoning skills. Based on their ratings of the alignment between the items and the intended constructs, we calculated the Content Validity Index (CVI), which yielded a value of 1, indicating high content validity. Table 1 provides an overview of the three constructs measured by the spatial reasoning test, along with the associated subjects, content, and items. Each item is labeled using the format “construct + number” (e.g., MR2 indicates that it is the second item of the test and it measures mental rotation). The test consists of 32 multiple choice items, scored on a 0–1 scale, with a maximum possible score of 32. Examples of items, along with their corresponding constructs and subjects, are presented in Table 2.

#### 2.3.2. Mathematics Test

Among the various assessments focusing on students’ mathematical performance, the Trends in International Mathematics and Science Study (TIMSS) is one of the most comprehensive international studies. This study takes the 32 released mathematical items for the fourth grade in TIMSS-2011 as the instruments, since the items of TIMSS-2011 are in the public domain and can be downloaded from the website of the IEA. The items of TIMSS-2011 are equipped with high reliability and validity and were still applied in recently published research (Hu et al. 2021; Xu et al. 2023). The mathematics assessment framework for TIMSS 2011 is organized around two dimensions: content and cognitive domains. There are three content domains described in the TIMSS 2011 Mathematics Framework for the fourth grade: number, geometric shapes and measures, and data display. The released items for the fourth grade in TIMSS-2011 are divided into six blocks (M01, M02, M03, M05, M06, M07). Considering the time constraints for students, this study selected three blocks—M01, M02, and M03—totaling 32 items as the test material, which include 17 items in the number domain, 11 in geometric shapes and measures, and 4 in data display. Some of these 32 items contained two or three sub-questions. Scoring adhered strictly to the TIMSS-2011 scoring guide, with a maximum possible score of 38: 20 for the number domain, 14 for geometric shapes and measures, and 4 for data display. Examples of items and their corresponding content domains are presented in Table 3.

### 2.4. Data Analysis

In the first phase, our aim was to validate the psychometric properties of the spatial reasoning test. The data analysis included evaluating the internal consistency of the test using Cronbach’s alpha, analyzing item difficulty and discrimination based on classical test theory, assessing construct validity through confirmatory factor analysis, and performing item analysis based on the Rasch model. In the second phase, the main study, to investigate the relationship between spatial reasoning and mathematics performance, regression analyses were conducted.

## 3. Results

### 3.1. Phase I: Instrument Analysis Results

#### 3.1.1. Reliability and Construct Validity

The internal consistency of the spatial reasoning test was evaluated using Cronbach’s alpha, yielding a result of 0.866 (>0.85), which indicates good reliability. The construct validity of the spatial reasoning test was evaluated using confirmatory factor analysis (CFA). As previously mentioned, the test was developed based on a three-tier framework of spatial reasoning ability. It is designed to measure overall spatial reasoning, but includes three constructs: mental rotation, spatial orientation, and spatial visualization. Accordingly, a second-order three-factor model was specified. As shown in Table 4, the fit indices from the CFA indicate a good model fit: the chi-square/df ratio is less than three, suggesting a well-fitting model; the root mean square error of approximation (RMSEA) is 0.030, below the 0.05 threshold, indicating excellent fit, with the upper limit of the 90% confidence interval at 0.035 also supporting good model fit; the standardized root mean square residual (SRMR) is 0.044, under the 0.08 threshold, reflecting an ideal model fit; and both the comparative fit index (CFI) and the Tucker–Lewis index (TLI) exceed 0.9 (0.918 and 0.912, respectively), further indicating that the model is acceptable. Therefore, based on these fit indices, the model demonstrates a good overall fit.

**Figure 1:**
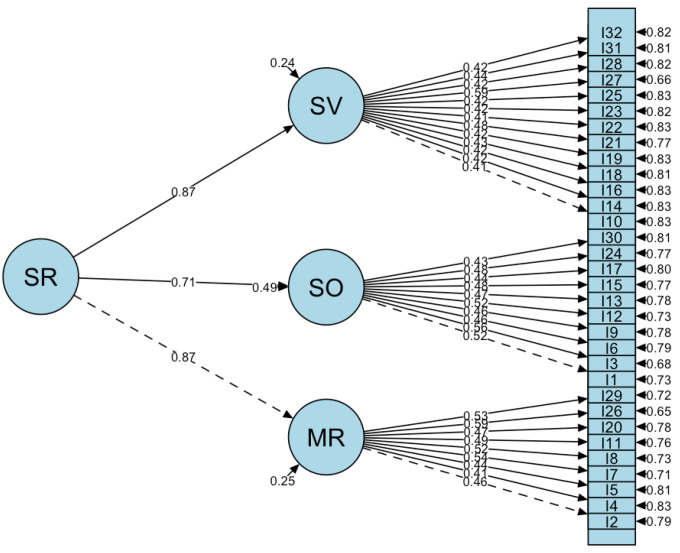
The second-order three-factor model of spatial reasoning.

Figure 1 illustrates the second-order three-factor model of spatial reasoning derived from the CFA. The standardized factor loadings of the items range between 0.413 and 0.587, with all *p*-values for the factor loadings being less than 0.01, and most being less than 0.001, indicating significant relationships between the first-order factors and their respective items. The factor loadings of the three first-order factors—mental rotation (MR), spatial orientation (SO), and spatial visualization (SV)—on the second-order factor, spatial reasoning ability (SR), are 0.868, 0.715, and 0.872, respectively, indicating strong correlations between the second-order factor and the three first-order factors. This suggests that all three constructs effectively measure spatial reasoning ability. Based on this analysis, the test demonstrates good construct validity.

#### 3.1.2. Item Difficulty and Discrimination

Table 5 presents the item difficulty and item discrimination indices based on classical test theory analysis. The overall difficulty level of the test items ranges from 0.27 to 0.92. Specifically, the difficulty level for mental rotation items spans from 0.46 to 0.81, for spatial orientation items from 0.46 to 0.92, and for spatial visualization items from 0.27 to 0.85. The discrimination indices fall between 0.29 and 0.52, all of which indicate medium-to-high levels of item discrimination.

#### 3.1.3. Item Analysis Based on Rasch Model

The Rasch model provides an objective standard for evaluating and refining assessment tools. Since unidimensional models in item response theory (IRT) are applicable only when a test measures a single latent trait, we first used principal component analysis (PCA) to test whether the spatial reasoning test conforms to the unidimensionality assumption. Before conducting PCA, we performed the Kaiser–Meyer–Olkin (KMO) measure of sampling adequacy and Bartlett’s test of sphericity. The KMO statistic was 0.89, falling within the “meritorious” range (0.8–0.9). Bartlett’s test of sphericity yielded a *p*-value of less than 0.001, well below the 0.05 threshold, indicating the data’s appropriateness for factor analysis (Dziuban and Shirkey 1974). In PCA, a ratio greater than three between the first and second eigenvalues generally supports unidimensionality. Our analysis revealed that the first component’s eigenvalue was 6.325, while the second component’s eigenvalue was 1.931, resulting in a ratio of approximately 3.28, which meets the unidimensionality assumption. Therefore, although the spatial reasoning test encompasses three constructs—mental rotation, spatial orientation, and spatial visualization—it primarily measures a single underlying structure: spatial reasoning.

The R-4.2.0 software offers various methods for parameter estimation, and this study employed the joint maximum likelihood estimation (JMLE) method, which simultaneously estimates both ability and item parameters. The person separation reliability was 0.845, indicating a high level of reliability (with values closer to one being preferable). The second column of Table 6 presents the item difficulty estimates, ranging from −2.989 to 1.259. Negative values indicate easier items, while positive values denote more difficult items. The mean item difficulty was −0.805, while the mean student ability was −0.040, suggesting that the overall test was slightly below moderate difficulty, with student ability slightly exceeding the test’s difficulty level. The third column of Table 6 shows the standard errors of the item difficulty estimates, which reflect the precision of these estimates—the closer to 0, the better. The standard errors ranged from 0.105 to 0.182, indicating that the estimation errors for item difficulty were relatively small.

Columns four through to seven in Table 6 present the fit statistics based on residuals for each item, including the mean square fit (MNSQ) and *t*-statistics. These statistics assess the degree to which the data fit the Rasch model. The Rasch model reports both outfit and infit MNSQ statistics. Outfit MNSQ, the unweighted fit mean square, is more sensitive to outliers, and thus more capable of identifying misfit issues. Infit MNSQ, the weighted fit mean square, mitigates the sensitivity to outliers, addressing the issue of certain items being misjudged as misfitting due to extreme values. An MNSQ value of one indicates perfect fit, values greater than one suggest underfit (where the data contain too much unexplained variance or noise), and values less than one indicate overfit (where the model overpredicts the data). The acceptable range for MNSQ indices is typically between 0.5 and 1.5, with values closer to 1 being ideal. As shown in Table 1, Table 2, Table 3 and Table 4, outfit MNSQ values range from 0.648 to 1.211, and infit MNSQ values range from 0.843 to 1.192, both of which fall within the acceptable range, indicating a good fit. Outfit *t* and infit *t* are the transformed *t*-statistics of the fit mean square values. These *t*-statistics only need to be further examined if the MNSQ indices fall outside the acceptable range (Boone et al. 2014). Generally, a reasonable range for the *t*-statistics is between −2 and 2. Apart from items 27 and 30, all items meet these criteria. Overall, the fit indices for the 32 items in this test indicate a good fit with the assumptions of the Rasch model.

### 3.2. Phase II: Main Study Results

#### 3.2.1. Descriptive Statistics

In this study, descriptive statistics were calculated for all eight variables, including overall spatial score, the three spatial reasoning constructs (mental rotation, spatial orientation, and spatial visualization), the overall mathematics score, and the three mathematics content domains (number, geometric shapes and measures, and data display). The means, standard deviations, range, skewness, and kurtosis for these variables are presented in Table 7. In line with the guidelines suggested by Kline (2016), if the absolute value of skewness is less than 3 and the absolute value of kurtosis is less than 10, the data can be considered approximately normally distributed.

The gender breakdown for these variables is provided in Table 8. Through independent samples *t*-tests, we compared females and males on the spatial and mathematics measures. No significant gender differences were found for performance in mathematics test, *t* = 1.27, *p* = 0.20 (>0.05). However, significant gender differences were found for performance in the spatial reasoning test, in favor of males, *t* = 2.60, *p* = 0.0096(<0.01). Further comparisons on the three spatial constructs and three mathematics content domains revealed gender differences in favor of males for mental rotation, *t* = 4.63, *p* < 0.001, and number, *t* = 2.52, *p* = 0.012 (<0.05); gender differences in favor of females for data display, *t* = −2.73, *p* = 0.006 (<0.01); no significant gender differences were found for spatial orientation (*t* = 1.44, *p* = 0.15), spatial visualization (*t* = 0.14, *p* = 0.89), and geometric shapes and measures (*t* = 0.34, *p* = 0.73). The findings of gender differences in overall performance on the spatial reasoning and mathematics tests, as well as in mental rotation tasks, align with previous research (Linn and Petersen 1985; Voyer et al. 1995; Hegarty 2018; Harris et al. 2021), However, the expected gender differences in spatial orientation reported in prior studies were not observed in this study (Logan and Lowrie 2017; Harris et al. 2021).

#### 3.2.2. Correlations Between Spatial and Math Variables

Table 9 summarizes the results of the bivariate correlations between all measured variables. Significant correlations were found between all spatial and mathematical measures (*p* < 0.001).

#### 3.2.3. Regression Analysis

Based on the strong correlations observed between spatial measures and mathematics measures, further regression analyses were conducted to explore the predictive relationships between spatial reasoning and mathematics performance. The analyses first aimed to identify the predictors of overall mathematics performance. Subsequently, predictors of performance in specific content domains, including number, geometric shapes and measures, and data display, were identified through separate regression models. In the multiple regression models, the collinearity statistics (i.e., VIF) were all within acceptable limits.

##### Predictors of Overall Mathematical Performance

As shown in Table 10, a simple linear regression indicated that overall spatial reasoning significantly predicts math performance (β = 0.66, *p* < 0.001), explaining 34.12% of the variance, with each point increase in spatial reasoning associated with a 0.66-point rise in math scores. A multiple regression analysis of three spatial constructs—mental rotation (MR), spatial orientation (SO), and spatial visualization (SV)—found that all significantly predict math performance (*p* < 0.001). Controlling for other variables, each one-point increase in MR, SO, and SV corresponds to 0.69, 0.67, and 0.64-point increases in math scores, respectively.

To further examine whether the impact of spatial measures on overall mathematics performance varies by gender, a subgroup regression analysis was conducted. Initial simple linear regressions for male and female students, as shown in Table 10, indicated that the overall spatial score had a significant positive effect on mathematics scores in both groups. Introducing the interaction term between spatial reasoning and gender showed no significant difference between groups (*p* = 0.982 > 0.05). Further multiple regression analyses for each gender group revealed that MR, SO, and SV positively impacted mathematics performance, with spatial orientation being the strongest predictor for males and mental rotation for females. The interaction terms for MR, SO, and SV with gender (*p* = 0.229, *p* = 0.059, *p* = 0.677) were not significant, indicating consistent effects across genders.

##### Predictors of Performance in Number

As shown in Table 11, simple linear regression reveals that overall spatial reasoning significantly predicts number performance, with each one-point increase in spatial reasoning corresponding to a 0.35-point increase in scores (β = 0.35, *p* < 0.001). The model explains 27.04% of the variance. Multiple regression analysis indicates that mental rotation (MR), spatial orientation (SO), and spatial visualization (SV) all significantly affect number scores (*p* < 0.001), with MR, SO, and SV predicting increases of 0.37, 0.41, and 0.29 points in number scores, respectively.

To assess if the predictive relationship between spatial reasoning and number performance varies by gender, subgroup regression analyses were conducted. Simple linear regressions revealed a significant positive effect of overall spatial score on number performance for both males and females. The interaction term for spatial reasoning and gender had a *p*-value of 0.706, indicating no significant differences in the effect across genders. Further multiple regression analyses showed that mental rotation, spatial orientation, and spatial visualization all significantly impact number performance in both genders. For males, spatial orientation was the strongest predictor, while for females, mental rotation was the most influential. Interaction terms for these spatial constructs with gender had *p*-values of 0.097, 0.312, and 0.658, respectively, suggesting no significant differences in their impacts on number performance between genders.

##### Predictors of Performance in Geometric Shapes and Measures

To assess whether spatial reasoning influences students’ performance in geometric shapes and measures, a simple linear regression was performed. As shown in Table 12, the overall spatial score significantly predicts geometric performance (β = 0.27, *p* < 0.001), indicating that each one-point increase in overall spatial score corresponds to a 0.27-point increase in scores. The model explains 30.75% of the variance. A multiple regression further revealed that mental rotation (MR), spatial orientation (SO), and spatial visualization (SV) all significantly contribute to performance in geometric shapes and measures. Specifically, MR (β = 0.32, *p* < 0.001), SO (β = 0.20, *p* < 0.001), and SV (β = 0.27, *p* < 0.001) each show positive effects on scores.

In the subgroup regression analyses, the impact of spatial reasoning on performance in geometric shapes and measures was examined for potential gender differences. Simple linear regressions for each gender group, as shown in Table 12, indicated that overall spatial scores significantly and positively influenced performance in geometric shapes and measures for both groups. Introducing an interaction term between spatial reasoning and gender revealed no significant difference between the groups (*p* = 0.896). Further subgroup multiple regressions showed that in the male group, mental rotation (MR), spatial orientation (SO), and spatial visualization (SV) all significantly predicted performance in geometric shapes and measures, with MR and SO having nearly equal impacts. In the female group, MR and SV were significant predictors, with MR being the stronger predictor, while SO did not significantly influence performance. Interaction terms between each spatial construct and gender indicated that SO’s impact significantly differed by gender (*p* = 0.02), while MR and SV’s impacts did not (*p* = 0.676 and *p* = 0.15, respectively).

##### Predictors of Performance in Data Display

To evaluate the influence of spatial reasoning on students’ data display performance, a simple linear regression was performed. The results, shown in Table 13, indicate a small but significant effect (β = 0.05, *p* < 0.001), with each one-point increase in overall spatial score corresponding to a 0.05-point rise in data display scores. Further analysis using multiple regression revealed that mental rotation did not significantly impact data display (*p* > 0.05), while spatial orientation (β = 0.06, *p* < 0.001) and spatial visualization (β = 0.08, *p* < 0.001) both had small, yet significant, positive effects.

Subgroup regression analyses were conducted to assess whether the impact of spatial measures on performance in data display differed by gender. Initial simple linear regressions for male and female students, as shown in Table 13, revealed that overall spatial score had a significant, but small, positive effect on performance in data display in both groups. The interaction term between overall spatial score and gender showed no significant difference in regression coefficients (*p* = 0.206). Further multiple regression analyses indicated that, while the predictors of performance in data display differed by gender—spatial orientation and spatial visualization were significant for males, and only spatial visualization was significant for females—the tests for coefficient differences between groups revealed no statistically significant differences (*p* = 0.948, *p* = 0.116, and *p* = 0.692, respectively), suggesting that the effects of these spatial constructs are consistent across genders.

## 4. Discussion

### 4.1. Summary of Findings

This study aimed to explore the role of spatial reasoning in predicting mathematical performance across different content domains among Chinese elementary school students, who have relatively limited exposure to space-related curriculum content. To achieve this, we administered a set of tests to students from four schools in three cities of China. We developed a spatial reasoning test based on a three-tier framework, adjusting existing instruments to ensure comprehensive coverage of each subject of the construct. This modified test was validated through preliminary analysis for quality and applicability among Chinese students in grades 4 to 6, providing a solid foundation for our study. Using this spatial reasoning test alongside TIMSS mathematics tests, we examined 816 fourth grade students to investigate the predictive relationship between spatial reasoning and mathematics performance. The findings demonstrate that despite the limited exposure to space-related content in the curriculum, spatial reasoning remains a significant predictor of mathematical performance among Chinese students, reinforcing the strong relationship between spatial reasoning and mathematics evidenced in prior studies (Frick 2019; Gunderson et al. 2012; Mix et al. 2016; Verdine et al. 2017). Specifically:

Overall Spatial Reasoning as a Predictor. The results indicate that overall spatial reasoning significantly predicts mathematical performance across various domains. This influence extends beyond the overtly spatial aspects of mathematics, such as geometric shapes and measures (Battista 2007; Clements and Battista 1992), to include the seemingly less spatial aspects of mathematics, such as the number domain, as highlighted in numerous studies (Hawes and Ansari 2020; Mix and Cheng 2012; Xie et al. 2020). Furthermore, spatial reasoning also significantly contributes to the data display domain, which is less-explored in relevant studies. Given that data literacy is regarded as one of the essential skills for citizens in the era of “big data” (Borges-Rey 2017), this finding may provide a new perspective on how spatial reasoning supports the development of data literacy. The significant impact of spatial reasoning on mathematical performance across all content domains in elementary school supports the notion that mathematics is inherently associated with spatial thinking (Congdon et al. 2018b; Jirout and Newcombe 2018).

Spatial reasoning constructs as predictors. All three spatial constructs—mental rotation, spatial orientation, and spatial visualization—significantly contribute to performance in the number and geometric shapes and measures domains. Mental rotation and spatial orientation emerge as the strongest predictors of performance in these respective domains. This finding contrasts with a previous study that found only object-based spatial constructs (mental rotation and spatial visualization) to be significant predictors (Harris et al. 2021). However, our results align closely with research indicating that egocentric transformations (e.g., mental rotation) showed the strongest relation to performance in arithmetic operations within the number domain, whereas allocentric transformations (e.g., spatial orientation) were strongly related to geometry (Frick 2019). The dominance of mental rotation (MR) in the number domain, even after controlling for spatial orientation (SO) and spatial visualization (SV), suggests that it uniquely captures dynamic egocentric transformations, such as mentally rotating numerical symbols (e.g., distinguishing ‘6’ from ‘9’) or manipulating quantities in working memory (Hawes et al. 2019). This persistent effect, despite the theoretical overlap between MR and SV, indicates that MR’s contribution is distinct and tied to real-time numerical transformations rather than stepwise spatial integration. Similarly, spatial orientation (SO) retained its strong predictive power for geometry after accounting for MR and SV. This aligns with its theorized role in allocentric perspective-taking, which is essential for decoding geometric diagrams (e.g., identifying congruent angles from different viewpoints). For the data display domain, both spatial orientation and spatial visualization significantly contribute, with spatial visualization being the strongest predictor. One possible explanation is that these constructs both involve decoding information, specifically interpreting graphic information, including visual elements and the spatial relationships among those elements within the graphics (Lowrie and Diezmann 2007; Lowrie and Logan 2018). When solving problems in the data display domain, students must recognize the various elements within a chart or graph and understand their relationships. For example, in the sample item of data display previously presented in Table 3, students are required to interpret visual elements, such as text or numbers in rows and columns, images of ice cream, and the spatial relationships between these elements and their corresponding mathematical meanings. The primacy of spatial visualization (SV) in predicting data display performance, even when MR and SO are controlled, likely stems from its reliance on schematic representations, which encode abstract spatial relations (e.g., chart layouts) rather than visual appearance (Hegarty and Kozhevnikov 1999). This representational advantage may enable students to efficiently extract, organize, and manipulate spatial information within complex graphical displays, facilitating mathematical reasoning in this domain. Notably, spatial orientation (SO) also uniquely contributed, potentially facilitating the spatial structuring of graphical layouts. However, its smaller effect size compared to SV suggests that SO may primarily aid in structuring spatial relationships within graphical displays, while SV plays a more central role in integrating and reasoning about these relationships.

Gender Differences. Although no significant statistical gender differences were found in the overall relationship between spatial reasoning and mathematical performance across content domains, subgroup regression analysis revealed variations. For male students, spatial orientation is the primary predictor of mathematical performance across content domains. For female students, however, mental rotation is the strongest predictor for number and geometry performance, while spatial visualization is the most significant predictor for data display performance. This pattern may be linked to differences in problem-solving strategies between genders. Prior research suggests that males tend to rely more on spatial strategies when solving mathematical problems, whereas females, even when presented with spatial tasks, may employ more verbal–analytical reasoning that does not require generating and manipulating mental images (Danan and Ashkenazi 2022). While this tendency does not necessarily lead to differences in overall math performance, it may influence which spatial constructs contribute most to mathematical success. In the present study, the stronger predictive role of spatial orientation for males aligns with the idea that they are more likely to engage in spatially based approaches to problem-solving. In contrast, the greater relevance of mental rotation and spatial visualization for females may reflect their tendency to approach mathematical tasks differently, potentially integrating verbal–analytical reasoning with specific spatial skills. This finding contrasts with Harris et al. (2021), who found that mental rotation and spatial visualization were more predictive of male math performance, while spatial orientation was more predictive of female math performance. The authors of that study suggested that this discrepancy might be due to a ceiling effect for males in spatial orientation tasks, potentially limiting the observed contribution of spatial orientation to math performance for males.

### 4.2. Educational Implications

Given the exceptional performance of students from East Asian countries in international mathematics assessments and the cultural context where space has received less emphasis in mathematics curricula compared to Western contexts (Lowrie et al. 2016), this study focuses on the spatial reasoning and mathematical performance of Chinese elementary school students, offering evidence from a different cultural perspective that further reinforces the strong relationship between spatial reasoning and mathematics. This highlights the importance of integrating spatial reasoning into elementary mathematics curricula as a fundamental component, particularly within the Asian mathematics curricula, where it is less heavily emphasized. To achieve this, it is crucial to encourage more non-Western researchers to focus on and expand the study of spatial reasoning, thereby enhancing reciprocal global research interactions.

By focusing on different mathematics content domains, this study clarifies the relationship between spatial constructs and mathematics, providing insights for more targeted educational interventions, particularly those that involve embedded interventions (Bruce et al. 2017; Davis and the Spatial Reasoning Study Group 2015; Hawes et al. 2017). This approach aims to further promote the spatialization of the mathematics curriculum, providing alternative ways that go beyond traditional approaches focused on computation, memorization, and repetition, for students to engage in mathematics (Mulligan 2015).

### 4.3. Limitations and Future Directions

This study investigated students’ spatial reasoning and their contribution to mathematics performance through a validated spatial reasoning test and the TIMSS mathematics test. However, several limitations should be noted. First, the participating schools were located in economically developed regions of China. Given that children from diverse economic backgrounds may rely on different skills and methods for problem-solving (Jordan et al. 1994; Butterworth et al. 2011), the generalizability of these findings to broader populations remains uncertain. Future research should include more economically diverse samples and examine the moderating effect of economic background on the relationship between spatial reasoning and mathematical performance. Second, as a correlational study, this research explored the predictive relationship between spatial reasoning and mathematics performance, but could not establish causality. Further intervention studies are necessary to explore the potential causal effects of spatial abilities on mathematical performance. Third, the current correlational analysis is based on test scores, which may not be fine-grained enough for deeper insights. Future studies might employ more sophisticated analytical methods that can better uncover latent traits. Additionally, the mathematics test was divided into three different content domains, with fewer items in the data display domain. This may have resulted in a less varied score distribution and ceiling effect, potentially impacting subsequent analyses. Future research focusing on the data display domain should consider using more comprehensive assessment tools. Finally, our study did not empirically compare the Chinese context with those which have a different curricular focus. Future studies could consider the link between spatial skills and mathematics in different educational settings.

## Figures and Tables

**Table 1:** Framework of the spatial reasoning test.

Construct	Subject	Content	Item
Mental Rotation (MR)	2D rotation	Determining the outcome of a rotation of a 2D object; differentiating between reflection and rotation, clockwise and anticlockwise turn	MR2, MR4, MR5, MR7, MR8, MR11
	3D rotation	Determining the outcome of a rotation of a 3D object; differentiating between reflection and rotation	MR20, MR26, MR29
Spatial Orientation (SO)	Orientation and location	Determining the position of an object in the situation; determining the position of the object relative to that of another object or the observer	SO1, SO3, SO6, SO12, SO17
	Alternate views	Front, top, or side view; identifying the orthogonal views of an object	SO9, SO15
	Navigating with maps	Moving and reorienting in a forward or inverted map according to the given route	SO13, SO24, SO30
Spatial Visualization (SV)	Part–whole relationships	Identifying parts from the whole and vice versa	SV14, SV18
	Reflection and symmetry	Finding the symmetry in an object; reflecting an object	SV3, SV4, SV5
	Folding and cutting	Visualizing the outcome of folding/unfolding/cutting a particular configuration; identifying cross sections of 3D objects	SV25, SV28, SV32
	Transformation between 2D and 3D	Constructing a 3D shape from a given 2D shape and vice versa	SV21, SV22, SV23, SV27, SV31

**Table 2:** Sample items in the spatial reasoning test.

Construct	Subject	Item
Mental Rotation (MR)	3D mental rotation	26. The diagram below represents a model made out of cubes. Which of the following is the same as the model? 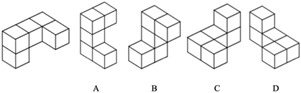
Spatial Orientation (SO)	Navigating with maps	24. A hamster was placed at the start of a maze, as shown below. The hamster ran through the maze. It turned to its right, then turned left, then turned right. Where did the hamster finish? 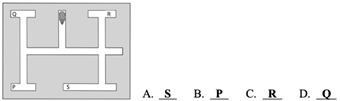
Spatial Visualization (SV)	Transformation between 2D and 3D	21. Diagram 1 represents a rectangular piece of paper. Which of the following hollow 3D shapes cannot be obtained by folding this rectangular paper? 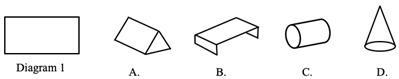

**Table 3:** Sample items in the mathematics test.

Item ID	Content Domain	Item
M051091	Number	Which fraction is not equal to the others? 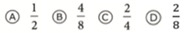
M051123	Geometric Shapes and Measures	How many lines of symmetry does this figure have? 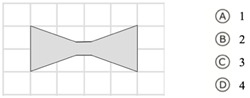
M051109	Data Display	How many children chose vanilla as their favorite flavor? 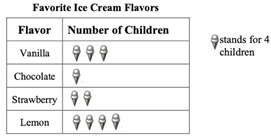

**Table 4:** Fit indices from the CFA.

	*χ* ^2^	*df*	*χ*^2^/*df*	CFI	TLI	RMSEA
Second-order three-factor model	653.664	461	1.418	0.918	0.912	0.030

**Table 5:** Item difficulty (*p*) and item discrimination (*D*) based on classical test theory analysis.

Mental Rotation			Spatial Orientation			Spatial Visualization		
Item	*p*	*D*	Item	*p*	*D*	Item	*p*	*D*
2	0.74	0.41	1	0.88	0.36	10	0.30	0.36
4	0.81	0.35	3	0.88	0.38	14	0.78	0.35
5	0.68	0.37	6	0.92	0.31	16	0.49	0.37
7	0.49	0.44	9	0.82	0.35	18	0.85	0.39
8	0.56	0.45	12	0.87	0.37	19	0.64	0.36
11	0.54	0.40	13	0.82	0.36	21	0.56	0.41
20	0.51	0.40	15	0.87	0.41	22	0.59	0.35
26	0.54	0.47	17	0.64	0.41	23	0.27	0.35
29	0.46	0.41	24	0.64	0.41	25	0.39	0.35
			30	0.46	0.29	27	0.37	0.52
						28	0.38	0.36
						31	0.66	0.35
						32	0.72	0.37

**Table 6:** Item information based on the Rasch model.

Item	Difficulty Estimate	SE	Outfit MNSQ	Outfit *t*	Infit MNSQ	Infit *t*
1	−2.413	0.150	0.662	−1.451	0.887	−1.224
2	−1.364	0.117	0.831	−1.209	0.950	−0.905
3	−2.391	0.149	0.648	−1.542	0.857	−1.593
4	−1.840	0.128	0.828	−0.926	0.962	−0.533
5	−0.978	0.110	1.072	0.645	1.011	0.248
6	−2.989	0.182	0.928	−0.105	0.866	−1.044
7	0.001	0.105	0.980	−0.230	0.976	−0.532
8	−0.342	0.105	0.927	−0.848	0.954	−1.075
9	−1.856	0.129	0.862	−0.714	0.960	−0.561
10	1.049	0.114	1.124	1.206	1.028	0.516
11	−0.224	0.105	1.012	0.177	1.009	0.214
12	−2.369	0.148	0.804	−0.776	0.843	−1.794
13	−1.856	0.129	0.894	−0.528	0.945	−0.789
14	−1.612	0.122	0.958	−0.204	0.973	−0.425
15	−2.287	0.145	0.653	−1.611	0.843	−1.881
16	0.022	0.105	1.066	0.854	1.066	1.504
17	−0.748	0.108	1.028	0.301	0.991	−0.188
18	−2.150	0.139	0.689	−1.528	0.891	−1.376
19	−0.759	0.108	1.004	0.070	1.055	1.196
20	−0.106	0.105	1.004	0.075	1.022	0.523
21	−0.331	0.105	0.994	−0.047	1.014	0.347
22	−0.504	0.106	1.077	0.847	1.072	1.629
23	1.259	0.118	1.098	0.881	1.042	0.707
24	−0.782	0.108	0.984	−0.119	0.994	−0.123
25	0.537	0.108	1.133	1.580	1.085	1.747
26	−0.256	0.105	0.899	−1.226	0.939	−1.418
27	0.628	0.109	0.785	−2.719	0.864	−2.907
28	0.616	0.109	1.088	1.044	1.069	1.410
29	0.184	0.106	0.972	−0.332	1.003	0.083
30	0.152	0.106	1.211	2.567	1.192	4.098
31	−0.850	0.109	1.034	0.342	1.065	1.372
32	−1.197	0.114	0.901	−0.740	1.011	0.238

**Table 7:** Descriptive statistics for eight variables.

	Mean	SD	Min	Max	Skewness	Kurtosis	SE
Spatial measures							
Overall spatial score	19.09	5.17	6	31	−0.02	−0.58	0.18
Mental rotation	5.11	2.28	0	9	−0.1	−0.8	0.08
Spatial orientation	7.38	1.8	1	10	−0.59	−0.08	0.06
Spatial visualization	6.6	2.3	1	13	0.01	−0.4	0.08
Mathematics measures							
Overall mathematics score	29.56	5.88	6	38	−1.09	1.13	0.21
Number	15.7	3.48	1	20	−1.06	1.11	0.12
Geometric shapes and measures	10	2.52	2	14	−0.75	0.14	0.09
Data display	3.46	0.79	0	4	−1.53	2.19	0.03

**Table 8:** Descriptive statistics for eight variables by gender.

	Male (N = 432)					Female (N = 384)				
	Mean	SD	Range	Skewness	Kurtosis	Mean	SD	Range	Skewness	Kurtosis
Spatial measures										
Overall spatial score	19.53	5.35	6–31	−0.08	−0.66	18.59	4.93	6–31	0.01	−0.5
Mental rotation	5.45	2.25	0–9	−0.27	−0.67	4.72	2.25	0–9	0.08	−0.81
Spatial orientation	7.46	1.82	1–10	−0.6	−0.1	7.28	1.78	1–10	−0.58	−0.07
Spatial visualization	6.61	2.48	1–13	0	−0.55	6.59	2.09	2–12	0.04	−0.31
Mathematics measures										
Overall mathematics score	29.81	5.95	7–38	−1.09	1.03	29.29	5.81	6–38	−1.1	1.25
Number	15.99	3.42	2–20	−1.12	1.25	15.38	3.51	1–20	−1	1
Geometric shapes and measures	10.43	2.51	2–14	−0.75	0.16	10.37	2.52	2–14	−0.75	0.1
Data display	3.39	0.84	0–4	−1.47	2.07	3.54	0.73	1–4	−1.54	1.82

**Table 9:** Correlations among the variables.

	1	2	3	4	5	6	7	8
Spatial measures								
1. Overall spatial score	-							
2. Mental rotation	0.82 ***	-						
3. Spatial orientation	0.76 ***	0.44 ***	-					
4. Spatial visualization	0.84 ***	0.51 ***	0.48 ***	-				
Mathematics measures								
5. Overall mathematics score	0.58 ***	0.48 ***	0.44 ***	0.49 ***	-			
6. Number	0.52 ***	0.43 ***	0.41 ***	0.42 ***	0.93 ***	-		
7. Geometric shapes and measures	0.55 ***	0.48 ***	0.39 ***	0.47 ***	0.86 ***	0.62 ***	-	
8. Data display	0.29 ***	0.18 ***	0.25 ***	0.29 ***	0.63 ***	0.52 ***	0.44 ***	-

*** *p* < 0.001.

**Table 10:** Predictors of overall mathematical performance.

Model		F	Adj. R^2^	β	SE	*t*
Overall Sample Regression (N = 816)						
Step 1: Simple Linear Regression						
Overall spatial score		421.5 ***	0.34	0.66	0.03	20.53 ***
Step 2: Multiple Regression						
		140.2 ***	0.34			
Mental rotation				0.69	0.09	7.74 ***
Spatial orientation				0.67	0.11	6.10 ***
Spatial visualization				0.64	0.09	7.09 ***
Subsample Regression: Male (N = 432) Female (N = 384)						
Step 3: Simple Linear Regression						
Male	Overall spatial score	240.8 ***	0.36	0.67	0.04	15.52 ***
Female	Overall spatial score	177.6 ***	0.32	0.66	0.05	13.33 ***
Step 4: Multiple Regression						
Male		80.89 ***	0.36			
	Mental rotation			0.59	0.12	4.77 ***
	Spatial orientation			0.86	0.15	5.75 ***
	Spatial visualization			0.60	0.12	5.11 ***
Female		60.69 ***	0.32			
	Mental rotation			0.81	0.13	6.17 ***
	Spatial orientation			0.45	0.16	2.78 **
	Spatial visualization			0.68	0.14	4.77 ***

*** *p* < 0.001; ** *p* < 0.01.

**Table 11:** Predictors of performance in number.

Model		F	Adj. R^2^	β	SE	*t*
Overall Sample Regression (N = 816)						
Step 1: Simple Linear Regression						
Overall spatial score		301.7 ***	0.27	0.35	0.02	17.37 ***
Step 2: Multiple Regression						
		101 ***	0.27			
Mental rotation				0.37	0.06	6.68 ***
Spatial orientation				0.41	0.07	5.98 ***
Spatial visualization				0.29	0.06	5.19 ***
Subsample Regression: Male (N = 432) Female (N = 384)						
Step 3: Simple Linear Regression						
Male	Overall spatial score	170.1 ***	0.28	0.34	0.03	13.04 ***
Female	Overall spatial score	126.3 ***	0.25	0.36	0.03	11.24 ***
Step 4: Multiple Regression						
Male		57.6 ***	0.28			
	Mental rotation			0.27	0.07	3.55 ***
	Spatial orientation			0.47	0.09	5.55 ***
	Spatial visualization			0.32	0.07	4.46 ***
Female		42.65 ***	0.25			
	Mental rotation			0.45	0.08	5.45 ***
	Spatial orientation			0.33	0.10	3.24 **
	Spatial visualization			0.27	0.09	2.98 **

*** *p* < 0.001; ** *p* < 0.01.

**Table 12:** Predictors of performance in geometric shapes and measures.

Model		F	Adj. R^2^	β	SE	*t*
Overall Sample Regression (N = 816)						
Step 1: Simple Linear Regression						
Overall spatial score		361.4 ***	0.31	0.27	0.01	19.01 ***
Step 2: Multiple Regression						
		121.5 ***	0.31			
Mental rotation				0.32	0.04	8.17 ***
Spatial orientation				0.20	0.05	4.22 ***
Spatial visualization				0.27	0.04	6.87 ***
Subsample Regression: Male (N = 432) Female (N = 384)						
Step 3: Simple Linear Regression						
Male	Overall spatial score	291.8 ***	0.34	0.273	0.02	14.83 ***
Female	Overall spatial score	145.7 ***	0.27	0.269	0.02	12.07 ***
Step 4: Multiple Regression						
Male		57.6 ***	0.34			
	Mental rotation			0.313	0.05	5.88 ***
	Spatial orientation			0.308	0.06	4.78 ***
	Spatial visualization			0.215	0.05	4.26 ***
Female		42.65 ***	0.28			
	Mental rotation			0.346	0.06	5.94 ***
	Spatial orientation			0.084	0.07	1.18
	Spatial visualization			0.331	0.06	5.24 ***

*** *p* < 0.001.

**Table 13:** Predictors of performance in data display.

Model		F	Adj. R^2^	β	SE	*t*
Overall Sample Regression (N = 816)						
Step 1: Simple Linear Regression						
Overall spatial score		77.15 ***	0.10	0.05	0.01	8.78 ***
Step 2: Multiple Regression						
		29.82 ***	0.10			
Mental rotation				0.00	0.01	0.12
Spatial orientation				0.06	0.02	3.43 ***
Spatial visualization				0.08	0.01	5.43 ***
Subsample Regression: Male (N = 432) Female (N = 384)						
Step 3: Simple Linear Regression						
Male	Overall spatial score	53.51 ***	0.11	0.05	0.01	7.32 ***
Female	Overall spatial score	29.31 ***	0.07	0.04	0.01	5.41 ***
Step 4: Multiple Regression						
Male		19.61 ***	0.34			
	Mental rotation			0.01	0.02	0.54
	Spatial orientation			0.09	0.02	3.42 ***
	Spatial visualization			0.07	0.02	3.40 ***
Female		11.31 ***	0.29			
	Mental rotation			0.01	0.02	0.49
	Spatial orientation			0.03	0.02	1.33
	Spatial visualization			0.08	0.02	3.78 ***

*** *p* < 0.001.

## Data Availability

The datasets used and/or analyzed during the current study are available from the corresponding author upon reasonable request.
